# Type III interferon exerts thymic stromal lymphopoietin in mediating adaptive antiviral immune response

**DOI:** 10.3389/fimmu.2023.1250541

**Published:** 2023-09-22

**Authors:** Luhong Cao, Weiwei Qian, Wanlin Li, Zhiyue Ma, Shenglong Xie

**Affiliations:** ^1^ Department of Otolaryngology Head and Neck Surgery Surgery, Sichuan Provincial People’s Hospital, School of Medicine, University of Electronic Science and Technology of China, Chengdu, China; ^2^ Department of Emergency Medicine, Laboratory of Emergency Medicine, West China Hospital, and Disaster Medical Center, Sichuan University, Chengdu, Sichuan, China; ^3^ National Clinical Research Center for Infectious Disease, Shenzhen Third People’s Hospital, Shenzhen, China; ^4^ Department of Thoracic Surgery, Sichuan Provincial People’s Hospital, University of Electronic Science and Technology of China, Chengdu, China

**Keywords:** type III interferon, thymic stromal lymphopoietin, IFN-III/TSLP axis, adaptive immunity, adaptive antiviral activity

## Abstract

Previously, it was believed that type III interferon (IFN-III) has functions similar to those of type I interferon (IFN-I). However, recently, emerging findings have increasingly indicated the non-redundant role of IFN-III in innate antiviral immune responses. Still, the regulatory activity of IFN-III in adaptive immune response has not been clearly reported yet due to the low expression of IFN-III receptors on most immune cells. In the present study, we reviewed the adjuvant, antiviral, antitumor, and disease-moderating activities of IFN-III in adaptive immunity; moreover, we further elucidated the mechanisms of IFN-III in mediating the adaptive antiviral immune response in a thymic stromal lymphopoietin (TSLP)-dependent manner, a pleiotropic cytokine involved in mucosal adaptive immunity. Research has shown that IFN-III can enhance the antiviral immunogenic response in mouse species by activating germinal center B (GC B) cell responses after stimulating TSLP production by microfold (M) cells, while in human species, TSLP exerts OX40L for regulating GC B cell immune responses, which may also depend on IFN-III. In conclusion, our review highlights the unique role of the IFN-III/TSLP axis in mediating host adaptive immunity, which is mechanically different from IFN-I. Therefore, the IFN-III/TSLP axis may provide novel insights for clinical immunotherapy.

## 1 Introduction

The interferon (IFN) response is a critical host defense mechanism against viral or microbial infections. According to sequence homology, IFNs can be subdivided into type I (IFN-I), type II (IFN-II), and type III IFNs (IFN-III). In humans and most mammals, IFN-I mainly include IFN-α, IFN-β, IFN-ϵ, IFN-κ, and IFN-ω; IFN-II group has only a single member, IFN-γ; IFN-III are the most recently discovered IFN family in 2003, which are composed of four isoforms including IFN-λ1 (IL-29), IFN-λ2 (IL-28A), IFN-λ3 (IL-28B), and IFN-λ4 (1). Humans have all IFN-III genes, while in mice, only the genes coding for *IFN-λ2* and *IFN-λ3* are present and *IFN-λ1* is a pseudogene (2, 3). *IFN-λ4* gene is the last to be discovered in human hepatocytes in 2013, but the genomic region encoding *IFN-λ4* is absent in mice (4). Although IFN-II is a key cytokine for establishing adaptive immunity and mediating pro-inflammatory and immunomodulatory features (5), IFN-I and IFN-III are more essential for innate immune responses, which is initiated by the recognition of pathogen-associated molecular patterns (PAMPs) via pattern recognition receptors (PRRs). In the context of virus invasion, viral DNA and RNA, as well as viral protein are sensed by PRRs, thus triggering the downstream signaling cascades to activate the production of IFNs from immune and non-immune cells (6, 7). Distinctly, IFN-I binds to cell surface receptors composed of two subunits IFNAR1 and IFNAR2, while IFN-III binds to the heterodimeric IFN-lambda receptor (IFNLR) composed of IFNLR1 and IL-10Rβ. After IFNs binding to their receptors, the Janus kinase/signal transducer and activator of transcription (JAK/STAT) signaling pathway is activated, which provides innate antiviral immune responses that depend on the expression of IFN-stimulated genes (ISGs). Ubiquitin-specific protease 18 (USP18) and suppressor of cytokine signaling 1 (SOCS1) are antagonisms of IFN-I, while IFN-III is less influenced by these negative regulatory mechanisms (8–12). Unlike IFN-I that is primarily produced by most immune cells, IFN-III is mostly secreted by epithelial cells and few immune cells like dendritic cells (DCs) (13). Additionally, although IFN-I receptors are ubiquitously expressed, IFNLR1 is preferentially expressed on the mucosal surface of epithelial cells rather than immune cells, except for neutrophils, which have recently been characterized as IFN-III responders (14–16).

Primarily, accumulating studies have provided evidence for the dominant role of IFN-I in mediating innate and adaptive immunity (17–21). Although IFN-λ has been discovered as a part of innate immune cytokines, its function in immune regulation remains elusive. The non-redundant role of IFN-λ in antiviral innate immunity has been widely summarized, such as host defense in the respiratory and gastrointestinal tracts, which can not be compensated by IFN-I (22–29). However, currently, little is known about the role of IFN-III in adaptive immune response. Over the past decade, the relevant research about the effect of IFN-III on adaptive antiviral immunity is limited, which may be due to the low expression of IFNLR in most immune cells (30), resulting in a limited understanding of the antiviral activity of IFN-III. With the further deepening of research, numerous reports regarding the responsibility of IFN-III in stimulating adaptive antiviral immunity continue to emerge (2, 16, 30–32). Particularly, the revealed mechanism of thymic stromal lymphopoietin (TSLP) unveils the mysterious role of IFN-III in orchestrating adaptive immune response (33–35). The assistance of TSLP in IFN-III-mediated immunoregulatory faculties triggers our interest in further research. Hence, this review will focus on the antiviral mechanisms of IFN-III in adaptive immune regulation, especially the IFN-III/TSLP axis in adaptive antiviral immunoregulation, as well as the differences between IFN-III and IFN-I in this antiviral immune system. Additionally, the inconsistent research conclusions between mouse and human species will also be discussed, yearning to provide novel viewpoints for vaccine development and clinical antiviral therapeutics.

## 2 IFN-III in adaptive immunity

### 2.1 Adjuvant activity

Adjuvants are substances added to vaccines to enhance the host’s antigen-specific immune response (36). The commonly used adjuvants (such as aluminum salts) could enhance immune response thought stimulating the NLRP3-inflammasomes and promoting the release of cytokines (such as IL-1β) (37). Other adjuvants (such as MF59 and AS03) exert functions by activating dendritic cells and other antigen-presenting cells (38). Additionally, some adjuvants interact with toll-like receptors (TLRs) or directly target specific immune cell populations. Cytokines, as important immunomodulatory agents, are available to affect any aspect of immune responses in a more or less specific manner (39, 40). As a newly described IFN family, the potential ability of IFN-III as a vaccine adjuvant to affect adaptive immune response has not been fully evaluated until an *in vivo* study shows that plasmid-encoded IL-28B can increase the virus-specific IgG2a titer in serum. This study reveals the significant potential benefits of IFN-III as an adjuvant in adaptive immune regulation. Moreover, IFN-III has been reported to further enhance splenic CD8^+^ T cell-mediated immunity by reducing regulatory T cell population during DNA vaccination (41). In another study, IL-28B adjuvant can remarkably elevate the antibody levels and neutralization titers of herpes simplex virus 2 in mice by DNA immunization. The IL-28B adjuvant-regulated immune response is significantly more effective than immunization with antigen DNA vaccine alone. Due to the involvement of IL-28B, T helper 1/2 (Th1/Th2) cytokines secreted by activated immune cells are crucial for controlling viral infection and disease progression (42). In addition, IFN-III subtype IFN-λ2 can also influence the production of antibodies against the influenza subunit vaccines. As a result, the influenza virus hemagglutinin (HA)-specific total serum IgG levels and its subclass IgG1 levels are about 15-fold enhanced. Besides, IFN-λ2 vaccination results in a significant increase in IgA levels in the bronchoalveolar lavage (BAL) fluids. Similar phenomenon can also be observed in another influenza vaccine CTA1-3M2e-DD (designated M2e) applied with IFN-λ2 (33). Obviously, IFN-λ2 and IFN-λ3 in mice can potentiate both cellular and humoral immune response in mice, representing promising molecular adjuvants. However, since the potential protection of IFN-III upon virus vaccination exhibits specificity on the mucosal surface, different immune approaches may affect the phenotype of immune responses (16, 22, 33). The characteristics of IFNLR enable IFN-III to serve as a significant front-line defense against viral infections at mucosal sites.

### 2.2 Adaptive antiviral effects

In addition to adjuvant activity, the IFN-III signaling also directly contributes to adaptive immunity against virus infection, which is mediated by direct stimulation of DCs or indirect effects on other immune cells like natural killer (NK) cells, monocytes/macrophages, T cells, and B cells to upregulate cytokines and chemokines (2, 8, 32). IFN-III can indirectly modulate the activation of NK cells and the inflammatory response of monocytes/macrophages, thus exerting auxiliary benefits in the antiviral activity of adaptive immunity against chronic hepatitis C virus (HCV) (43). The common single-nucleotide polymorphisms (SNPs) in the IFN-III signaling are strongly associated with the treatment of chronic HCV infection (31, 32). In the context of yellow fever virus infection, IFN-III deficiency leads to impaired T cell activation and severe perturbation of pro-inflammatory cytokines in mice (44). In response to lymphocytic choriomeningitis virus (LCMV) infection, IFN-III increases CD8^+^ T cell expansion and diminishes weight loss in the chronic pathological stage instead of the acute infective phase, indicating that virus-specific host interactions affect the regulatory mode of IFN-III in the adaptive immune response (45). Further researches investigate the critical role of the IFN-III signaling in combating different subtypes of influenza A virus (IAV) strains (PR8 and X31), which is orchestrated through indirect adaptive immune mechanisms. The adaptive immunomodulation does not rely on the direct attachment of IFN-III to CD8^+^ T cells, but rather on IFN-III triggering DCs, particularly manipulating the migration and function of CD103^+^ DCs, which is essential for optimal antigen uptake and CD8^+^ T cell activation, thus providing permanent IAV protection (46). The activation program of DCs is probably regulated by TSLP, but it has not been confirmed yet.

### 2.3 Anti-tumor effects

IFN-III has been demonstrated to possess additional benefits in suppressing tumors in mice by directly reducing the tumorigenicity of tumor cells, inhibiting cell proliferation, and inducing apoptosis (47–49). IFN-III also mediates indirect anti-tumor mechanisms by regulating the tumor microenvironment (50). NK cells and CD8^+^ T cells are recruited into the microenvironment to participate in the anti-tumor mechanism of IFN-III in a murine model of melanoma (51). A murine model of fibrosarcoma further confirms the indirect anti-tumor mechanism of IFN-III. *In vivo*, antibody depletion of NK cells, neutrophils, and CD8^+^ T cells, but not CD4^+^ T cells, induces IL-28-mediated inhibition of tumor growth. Meanwhile, the inoculation of IL-28-encoded fibrosarcoma cell line in mice advances the production of IFN-γ and evokes the activity of cytotoxic T cells, indicating that the anti-tumor action of IL-28 also partly depends on IFN-γ (52). The IFN-III signaling can target mammary epithelial cells and promote the chemokine CXCL10 production, thereby recruiting CD4^+^ T cells into the tumor microenvironment. Besides, the immune balance between Th1 and Th2 altered by IFN-III may take advantages in the treatment of cancer (53). In addition, IFNLR-deficient (*Ifnlr^-/-^*) mice are more sensitive to experimental tumor metastasis and carcinogen-induced tumor formation and are also more susceptible to the growth of NK cell-sensitive lymphoma. Administration of IFN-III postpones the fatality in the B16F10 melanoma model mice with adoptive cell transfer and diminishes tumor growth in methylcholanthrene-treated mice (54). Therefore, IFN-III may be utilized as an adjuvant anti-tumor therapy with its ability to modulate tumorigenesis directly and indirectly. Due to the lower expression of IFNLR1 in most cell types, IFN-III offers a more selective targeted anti-tumor ability with fewer side effects compared to IFN-I (55).

### 2.4 IFN-III in hypersensitivity disorder

#### 2.4.1 Autoimmune disease

IFN-III was initially described as a specialized system that inhibits viral replication on the surface of the epithelial barrier while limiting inflammatory damage. However, although IFN-III has complex effects on both innate and adaptive immunity, it is also associated with systemic autoimmune diseases (56). IFN-III exerts protective activity in chronic skin diseases such as psoriasis by secreting CXCL10 and CXCL11, to attract NK cells, CD4^+^ and CD8^+^ T cells. Elevated IFN-III in human keratinocytes contributes to relieving psoriatic lesions (57). Importantly, IFN-III demonstrates its heterogeneous characteristics in autoimmune rheumatic diseases. Compared with healthy controls, patients with systemic lupus erythematosus (SLE) have higher serum levels of IFN-III (58), which is also correlated with the upregulation of inflammatory cytokines including IL-6, IL-27, Th17, and B cell activating factor (BAFF) (59–61). Data from TLR7-induced lupus *Ifnlr* ^-/-^ mouse models support the mechanistic role of IFN-III in SLE. The infiltration of CD4^+^ and CD8^+^ effector memory T cells, B cells, macrophages, and neutrophils in *Ifnlr* ^-/-^ mice are less than that in wild-type (WT) mice, which contributes to weakening the over-activated immunity and diminishing inflammation in the skin and kidneys (62). These data suggest that IFN-III may promote the autoimmune pathology of SLE. The serum level of IFN-λ1 has also been found to increase in rheumatoid arthritis (RA) patients (63), and the pro-inflammatory cytokines like IL-6 and IL-8 are further increased in synovial fibroblasts after IFN-λ1 application (64). However, another research demonstrates the protective effect of IFN-λ2 in mice with collagen-induced arthritis (65). Treatment with IL-28A effectively reverses the development of arthritis by reducing IL-17 levels in joints and inguinal lymph nodes after restricting the recruitment of IL-1β-expressing neutrophils (65). Therefore, whether IFN-III is the culprit of RA remains unclear. Furthermore, IFN-III also contributes to the pathogenesis of autoimmune disease in the central nervous system (CNS). In an experimental autoimmune encephalomyelitis (EAE) murine model of multiple sclerosis (MS), *Ifnlr* ^-/-^ mice are associated with diminished Th1 cells and decreased pro-inflammatory cytokines compared with WT animals. The neutralization of IFN-III signaling also promotes the recovery of autoimmunity in the CNS (66). Taken together, these data highlight the intimate relationship between IFN-III and autoimmune diseases.

#### 2.4.2 Asthma

For hosts with asthma, IFN-III has been reported to hamper hypersensitized immunity. In experimental allergic models, the application of IFN-III can not only reduce granulocyte recruitment in BAL and inflammation in lung tissues (67) but further modulate TH1 and TH2 responses in a dose-dependent manner (68). Additionally, most patients suffer from asthma exacerbation after virus infection. For that, IFN-III production stimulated by the virus in the lung exerts antiviral effects without triggering inflammation (69). IFN-III has been evidenced to decrease IL-4, IL-5, and IL-13 production in asthma, which also provides protective advantages in hypersensitivity disorder (70). However, the role of IFN-III varies in patients of different ages and with different disease stages; for instance, IFN-III level is generally stable in stable asthma in adults, which represents more effective disease control (71).

## 3 TSLP in mucosal adaptive immunity

TSLP is a short-chain hematopoietic cytokine that signals through TSLP receptor (TSLPR), a high-affinity heterodimeric receptor composed of IL-7α-chain and a unique TSLP chain (72). TSLP is predominantly produced by epithelial cells in mucosal tissue (73). Additionally, TSLP can also be produced by other immune cells (such as fibroblasts, DCs, monocytes, and mast cells) following stimulation (74). In most studies, TSLP is described as a pathogenic cytokine that can trigger allergic diseases (such as allergic dermatitis and asthma) (75) and induce pro-inflammatory factors in autoimmune diseases (76–78), particularly in barrier tissues (such as skin, lungs, and intestines) (79). TSLP can activate DCs to promote a Th2-type immune response associated with allergy disease. Moreover, TSLP also affects regulatory T cells (Tregs) development, contributing to immune tolerance (80, 81).

However, TSLP also performs a non-redundant role in antiviral, antibacterial, and antitumor regulation to some extent. TSLP interacts with TSLPR on CD11b^+^ DCs to participate in immune responses and promote the long-term antiviral immunity of CD8^+^ T cells following influenza infection in mucosal tissues, especially in the respiratory tract (82, 83). The TSLP-TSLPR pathway in CD11c^+^ cells is also essential for inducing respiratory antigen-specific IgA in response to pneumococcal infection (84). Moreover, TSLP can potently induce adaptive immunity mediated by CD4^+^ Th2 cells to block the early carcinogenesis of breast cancer without causing inflammatory responses in normal breast tissues (85). However, recent researches have also suggested that TSLP might have both pro-tumorigenic and antitumor effects, and its activity depends on the tumor microenvironment context and the cancer type (74, 86).

As a B cell-associated cytokine, TSLP not only performs immune activity via direct ingestion but also serves as a vaccine adjuvant (87). With the participation of TSLP as an enhancer, TSLP-activated DCs can induce much robust CD8^+^ T cell expansion, thus providing more efficient anti-tumor immunotherapies than historically used adjuvant Bacillus Calmette–Guérin (88). As an adjuvant of the human immunodeficiency virus (HIV) vaccine CN54gp140, intranasal inoculation of TSLP, rather than intradermal inoculation, triggers potent and sustained serum and mucosal immune responses without inducing additional inflammatory responses. With the aid of TSLP, DCs located in the nasal mucosa are activated and Th2-cell-skewed immune responses are strongly promoted, followed by B cell differentiation and antigen-specific IgA and IgG1 secretion (89). The combination of TSLP and IAV-M2e vaccine can damper the weight loss of mice, and all animals can survive during the challenge infection period. Furthermore, TSLP stimulates an increase in serum M2e-specific IgG1 levels. The same results can be observed when TSLP is given together with another IAV vaccine haemagglutinin (HA), a commercial influenza vaccine containing particle-derived hemagglutinin. In TSLPR deficient (*Tslpr* ^-/-^) mice, intranasal immunization with the IAV vaccine fails to boost neither antigen-specific IgG1 nor nucleoprotein-specific CD8^+^ T cells compared with WT mice (33, 35). Taken together, these data verify the non-redundant role of TSLP in mucosal adaptive immunity, whether administrated alone or combined with vaccination.

## 4 IFN-III mediates adaptive immunity via TSLP

### 4.1 Importance of the IFN-III/TSLP axis in adaptive immunity

**Figure 1:**
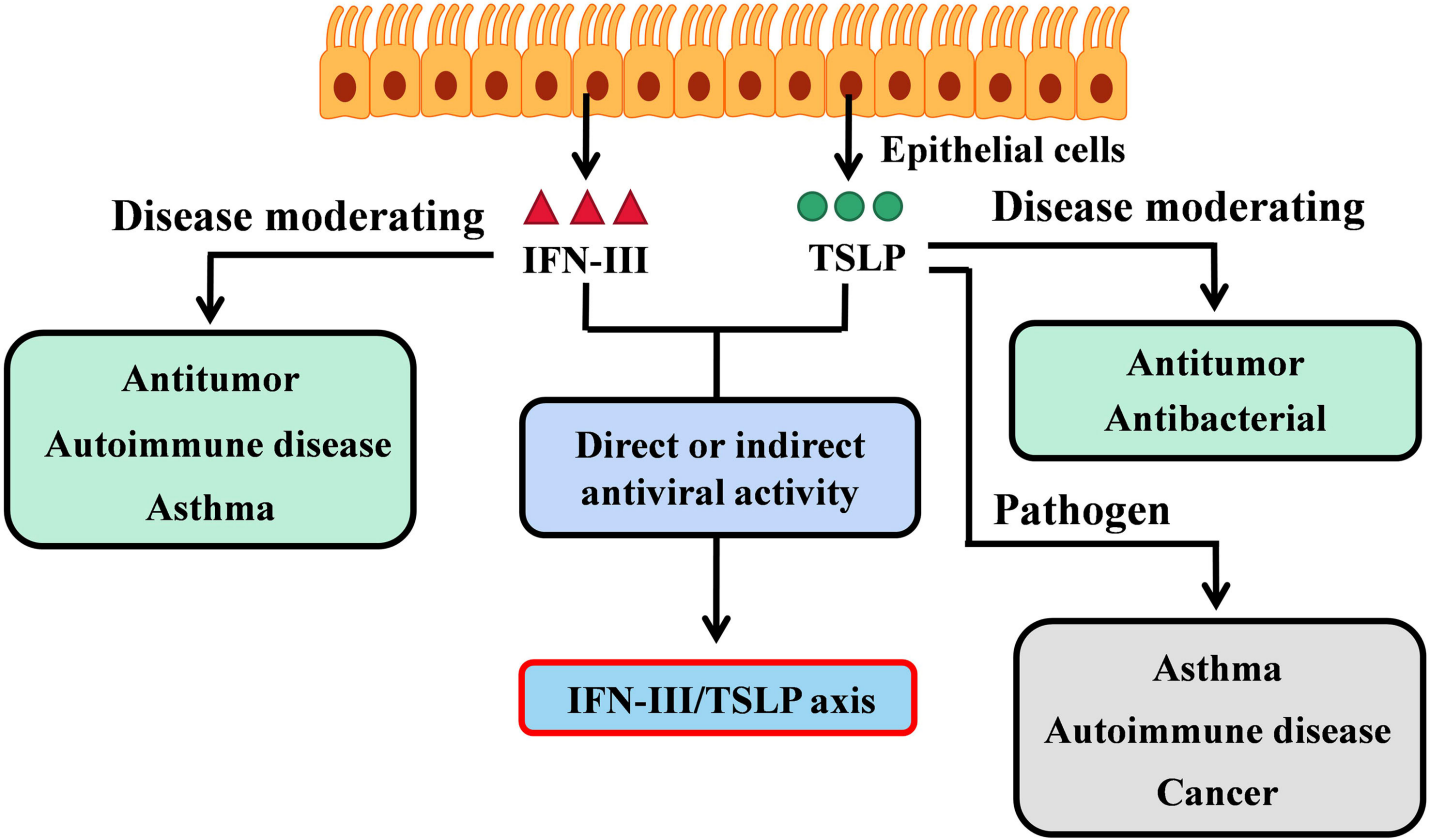
The IFN-III/TSLP axis in adaptive immunity. IFN-III and TSLP are both produce by epithelial cells, but perform different biological activities. IFN-III is critical in modulating tumor invasion or hypersensitivity disorders (such as autoimmune disease and asthma); TSLP is the pathogen of asthma and autoimmune disease but contradicts in cancer. Interestingly, both IFN-III and TSLP show direct or indirect antiviral activities on mucosal sites. The interaction between IFN-III and TSLP has also been revealed by the latest researches (33, 35). Therefore, the IFN-III/TSLP axis is responsible for adaptive antiviral immunity but is unclear in the context of cancer or hypersensitivity disorder.

Considering that both IFN-III and TSLP are produced by epithelial cells and both play roles in immune response as described above, it is of great significance to investigate the relationship between these two cytokines. Current studies have shown that intranasal inoculation of influenza virus subunit vaccine containing TSLP can effectively reinforce the serum IgG1 levels to a similar extent as IFN-λ2 triggered. However, in *Tslpr* ^-/-^ mice, immunization with M2e or HA-based Influsplit Tetra IAV vaccine combined with IFN-III as an adjuvant inhibits the enhancing effect of IgG1, indicating that TSLP can replace the enhancing activity of IFN-III, and TSLP is required for IFN-III-induced immune protection. Simultaneously, the losing function of TSLP in the IAV vaccine in *Tslpr-/-* mice can’t be compensated even in the presence of IFN-λ2 (33). These results indicate that the positive effects of IFN-III on the production of IgG1 and IgA depend mechanistically on TSLP. Interestingly, thus TSLP-mediated protective antiviral immunity of the IFN-III can similarly be realized through rectal vaccine inoculation. Whereas the stimulating effect of IFN-III as an adjuvant in influenza vaccine is canceled if administered intraperitoneally or subcutaneously, implying that these non-mucosal sites do not express significant IFN-III-responsive cells to produce TSLP-dependent antibodies (35). Taken together, we proved that TSLP was responsible for the IFN-III-related adaptive immune system. The IFN-III/TSLP axis (refer to some immune interactions between TSLP and IFN-III) participated in antiviral adaptive immune regulation at mucosal sites, particularly in the respiratory and gastrointestinal tracts (**Figure 1**).

Although IFN-III and TSLP are both produced by epithelial cells and exert effects on antiviral and antitumor immune regulation, a direct or well-defined interaction between the two in moderating cancer or hypersensitivity disorder is still lacking. Nevertheless, based on adaptive immunity exerted by the IFN-III/TSLP axis in the context of virus infection, there remains an area of ongoing research.

### 4.2 The IFN-III/TSLP axis is mechanistically different in mouse and human

**Figure 2:**
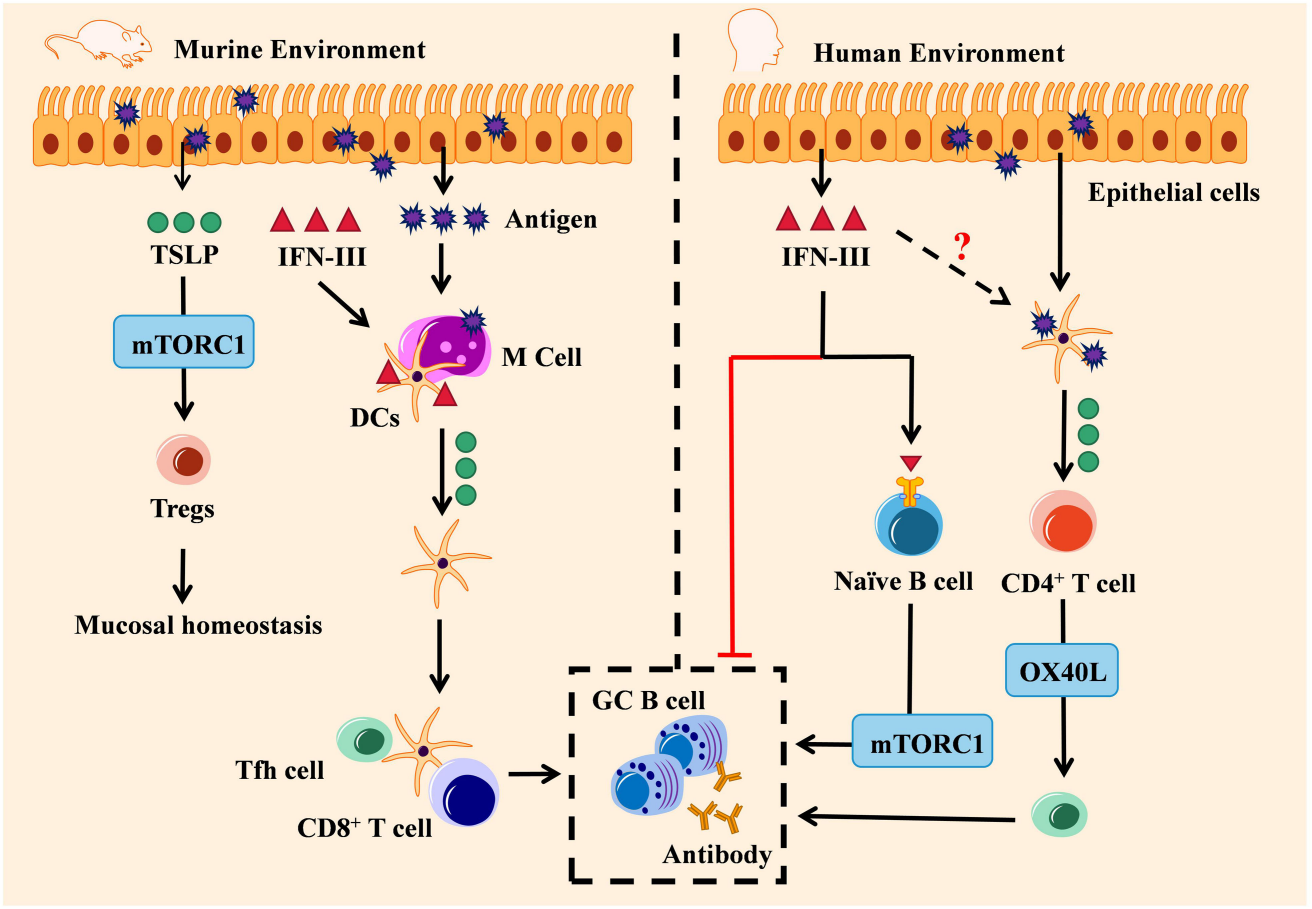
Engagement of the IFN-III/TSLP axis in adaptive antiviral activity: environment decided. The IFN-III/TSLP axis exerts distinct adaptive antiviral activities in mice and human. In the murine environment (left), IFN-III induces TSLP production by virus-infected M cells and promotes the migration of CD103^+^ DCs, thereby promoting TSLP-dependent GC B cell response by Tfh cells and fostering high-affinity antibody production. Antigen-stimulated TSLP also regulates mucosal homeostasis by activating Tregs through the mTORC1 pathway. In the human environment (right), on the one hand, IFN-III can either inhibit antibody production or enhance GC B cell response by naïve B cells through the mTORC1 pathway; on the other hand, TSLP-activated DCs are capable of inducing naïve CD4^+^ T cells to differentiate into Th2 via the OX40L pathway, and this process may also depend on IFN-III.

IFNLR is expressed in human B cells while lacking in mice, which suggests that the IFN-III/TSLP axis may have different immunoregulation functions in mice and humans (14). As mentioned previously, TSLP is a cytokine modulating antibody production towards IgA and IgG via the intranasal route (89). IFN-III vaccination in mice infected with the influenza virus can strongly trigger microfold (M) cells on the upper respiratory tract, which induces TSLP production, CD103^+^ DCs migration, and promotes the production of follicular helper T (Tfh) cells in lymph nodes. Later, TSLP-dependent germinal center (GC) B cells in lymph nodes are activated and thus numerous high-affinity IgA and IgG antibodies are secreted (33, 90). Besides, apart from the critical role of IFN-III in mucosal homeostasis (91), TSLP has also been found highly expressed in antigen-stimulated colonic epithelial cells, which in turn activates Tregs through the mTORC1 pathway to maintain mucosal homeostasis (92). Hence, the IFN-III/TSLP axis may also participate in modulating mucosal homeostasis to some extent (**Figure 2**, left).

In the human species, the IFN-III/TSLP axis seems to show inconsistent roles. A previously *in-vitro* study has indicated that IFN-III is associated with a decrease in Th2-cytokines, B-cell proliferation, and IgG production in IAV-infected patients (93). By contrast, another study has shown that human naïve B cells interact with IFN-III stimulation and then differentiate into plasmablasts through the mTORC1 pathway (94). In respiratory epithelial cells, human *Tslp* genes are expressed during respiratory syncytial virus infection (95). TSLP-activated DC cells can induce Tfh cells to differentiate from naïve CD4^+^ T cells into a Th2-skewed environment, mechanistically depending on OX40L, a ligand of OX40 (96). Since previous *in vitro* studies have demonstrated that human TSLP can modulate GC-B cells’ immune response and B cells from humans directly respond to IFN-III (94, 97), it is currently unknown whether the coordination of the IFN-III/TSLP axis in adaptive antiviral immunity can also be replicated in humans, as observed in mice (**Figure 2**, right).

The reason why IFN-III-stimulated B cells show completely different functions between humans and mice is currently unclear. However, there are indications that the IFNLRI gene in various cell types is correlated with epigenetic changes, including DNA methylation and histone modifications. As has been evidenced previously, the transcriptional regulation of the IFNLR1 gene is dynamic, which may influence IFNLR1 expression and result in multiple functions. Indeed, histone deacetylase inhibitors can cancel the silencing of the IFNLR1 gene and confer IFN-III responsibility in previously inactivated human cell lines. The dynamic expression of IFNLR may explain the conflicting role of IFN-III in adaptive viral protection (98).

Considering that the *in-vivo* mouse models and *in-vitro* human studies cannot completely demonstrate the exact operation of IFN-III in the human body, a more relevant model on non-human primates may exhibit closer effects. Interestingly, under IFN-λ3 stimulation, macaques exhibit an adaptive immune response consistent with the immune activity in mice. In rhesus macaques, DNA vaccination encoding IFN-III exerts a positive effect on enhancing the antiviral activity of antigen-specific CD8^+^ T cells. Moreover, IFN-λ3 adjuvant can further induce continuous Th1 cell-biased immune responses (99, 100).

### 4.3 IFN-I orchestrates adaptive immunity via a TSLP-independent mechanism

IFN-I can promote adaptive immune response or possess adjuvant activity against virus infection, bacterial burden, and tumor invasion (18, 101–105). The role of IFN-I in autoimmune diseases is bifacial. Though IFN-I is correlated with severe SLE progression, it may also play a positive role in the pathological improvement of RA (106, 107). Here we focus on the adaptive antiviral activity of IFN-I.

**Figure 3:**
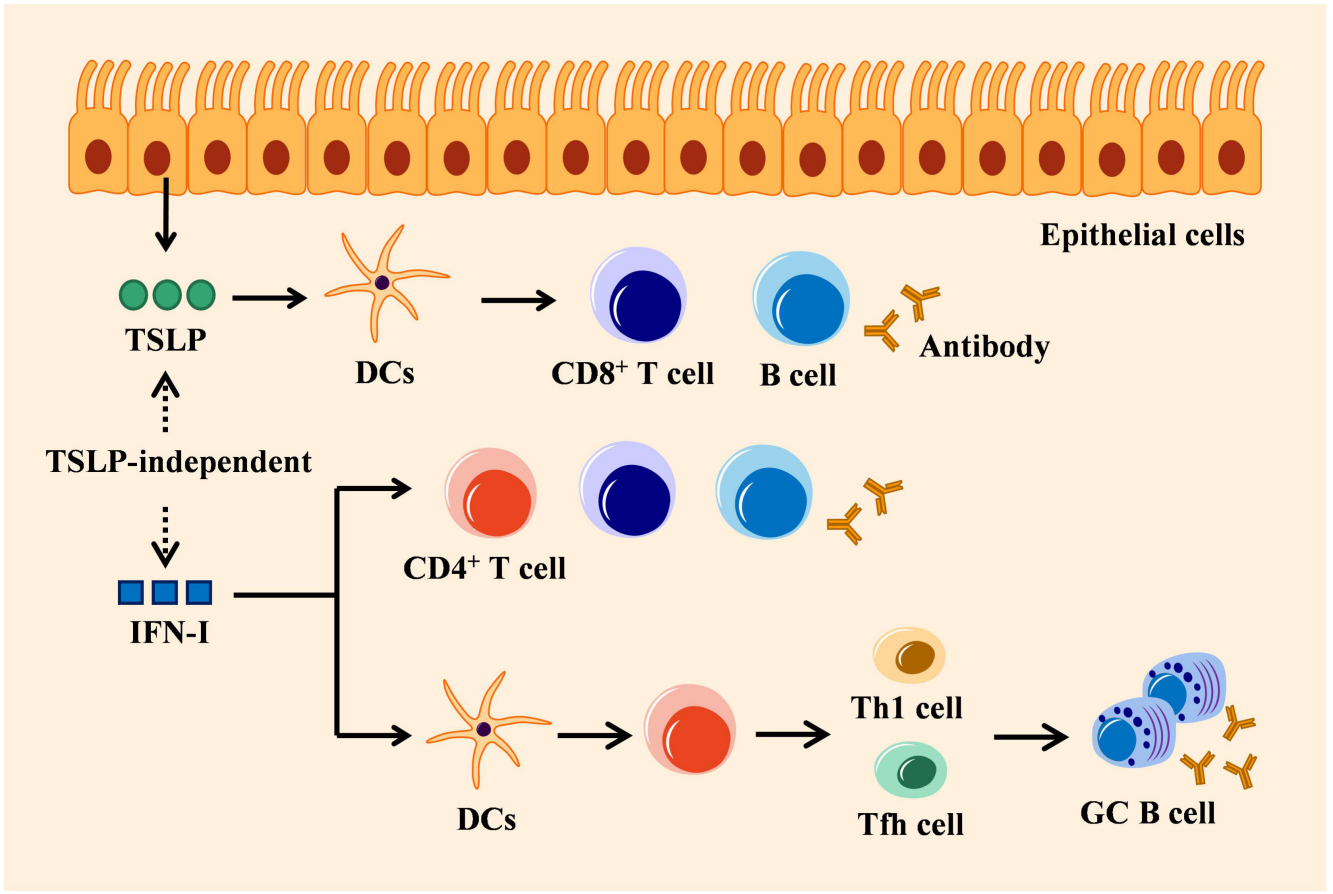
Mechanisms of IFN-I in adaptive immunity: TSLP-independent. Epithelial cells-derived TSLP exerts adaptive antiviral activity by activating DCs that trigger CD8^+^ T cell amplification, B cell differentiation, and antigen-specific antibody secretion. IFN-I regulates adaptive immune response directly by stimulating immune cells (such as T cells or B cells), which is independent of TSLP, or indirectly by stimulating DCs to induce CD4^+^ T cells to differentiate into Tfh or Th1 cells that can activate antibody production by GC B cells.

IFN-I and IFN-III can commonly be secreted by most nucleated cells, while IFN-III is particularly secreted by epithelial cells of mucosal organs. Although the signaling pathways triggered by these two types of IFNs are similar, their adaptive antiviral immune responses display functionally different (8–12). IFN-I modulates adaptive immunity via direct or indirect mechanism that depends on its impact on B and T cells involved in the adaptive immune response. In indirect mechanism, IFN-I firstly binds to DCs expressing IFNAR (108), thereby stimulating DCs to produce chemokines and cytokines that enhance the survival and proliferation of T cells including CD4^+^ T cells and CD8^+^ T cells, as well as induce the differentiation of T helper cell subsets including Th1 and Tfh cells (109–111). The activated Tfh cells migrate to B cell follicles and participate in GC reactions to generate high-affinity antibodies. Th1 cells on the other way express the master transcription factor T-bet and produce high levels of IFN-γ, which promotes macrophage activation and enhances CD8^+^ T cell responses. However, the effect of IFN-I on CD4^+^ T cell differentiating into Tfh and Th1 may disparate, which depends on the cells sensing these cytokines, the nature of the pathogen, and the stage of the infection (112–115). IFN-I signaling also regulates B cell response indirectly by inducing DCs to secret B cell stimulatory cytokines, thereby promoting immunoglobulin (Ig) isotype switching (116). This pathway acts on the mediation of IFN-I between CD4^+^-differentiated Th1 and Tfh to prosper GC B cell response (117). In the lungs, IFN-I-induced CXCL13 can initiate CXCR5-dependent recruitment of B cells and support ectopic GC formation (118). In direct adaptive immune regulation, IFN-I signaling immediately activates T cell receptor, thus promoting T cell proliferation and differentiation (110). IFNAR-deficient B cells induce an impaired antibody response and subclass switching when treated with IFN-α, indicating a direct effect of IFN-I on B cells (119). Indeed, IFN-I is required for different stages of B cell response, including early B cell activation and GC formation (120). Unlike IFNAR that is extensively expressed on most hematopoietic and non-hematopoietic cells, IFNLR is absent on the surface of mouse B and T cells (30), but specifically activated on the epithelial surface (16). Hence, IFN-I exhibits an adaptive immune response directly by interacting with immune cells, instead of mucosal epithelial cells, which is completely different from IFN-III (**Figure 3**).

Although IFN-I and IFN-III are engaged in almost the same intracellular signaling pathway, it is of interest that, the functions of IFN-I and IFN-III in their adjuvant activities for influenza vaccines are different. On the one hand, when the influenza vaccine is applied with IFN-αB/D intranasally, both the antigen-specific IgG1 and IgG2c levels in serum and the IgA levels in BAL fluids are elevated. IFN-I can also improve vaccine-induced serum antibodies after intraperitoneal immunization. In contrast, IFN-λ2-enriched influenza vaccine promotes the IgA levels in BAL fluids and IgG1 levels in serum but not IgG2c. Thus IFN-III-induced vaccine immune responses are not observed either through intraperitoneal or subcutaneous routes. On the other hand, while the immune activity of IFN-λ2 plus vaccine is dampened in *Tslpr* ^-/-^ mice, antibodies in both serum and BAL fluids remain unaffected in TSLP-deficient mice administrated with IFN-αB/D-involved influenza vaccine (33, 35). In summary, the adjuvant activity of IFN-I is not restricted to mucosal tissue. The vaccine-induced influenza virus resistance mediated by IFN-I does not rely on TSLP immune regulation. On the contrary, TSLP is important for IFN-III-mediated adaptive immune response (34). Hence, IFNs can be selectively used in the application of clinical adjuvants. IFN-III may be more suitable for mucosal immune regulation and epithelial surface protection, whereas IFN-I provides more benefits in systematical immune responses (121). Therefore, the selective use of IFNs contributes to efficient and targeted immunotherapy.

## 5 Discussion

The studies we mentioned above have revealed the non-redundant role of IFN-III and TSLP in adaptive immune modulation respectively. Moreover, the vital role of the IFN-III/TSLP axis in adaptive response was further highlighted. As described, the adaptive immune regulatory faculty of IFN-III is indirect and depends on TSLP. IFN-III triggers TSLP synthesis from M cells specialized in the upper respiratory tract; TSLP then promotes the maturation and migration of CD103^+^ DCs to draining lymph nodes, where DCs stimulate CD8^+^ T cells from naïve state and activated GC B cells by affecting Tfh cells, thus producing virus-specific IgG1 and IgA (33, 35). With the participation of adjuvants, vaccine-induced protective immunity can achieve stronger immune responses than antigens alone (122). In the mouse models of influenza virus vaccination, adaptive antiviral immune responses are markedly dependent on the previously unknown IFN-III/TSLP axis, which is not found in IFN-I adjuvant activity (34). Cytokines, as natural peptides closely related to the host immune response, have unique advantages as potential vaccine adjuvants (123). We herein highlighted the crucial function of IFN-III and TSLP cytokines, as well as the IFN-III/TSLP axis in modulating adaptive immune responses.

Nevertheless, the potential function of the IFN-III/TSLP axis in modulating adaptive immunity has not been fully explained. On the one hand, limited by the genetically different IFN-III system between humans and mice, which IFN-III subtypes confer immune activity in the IFN-III/TSLP system in both humans and mice remains unclear. On the other hand, our review shows that IFN-III modulates mucosal adaptive immunity via TSLP in mice; however, considering the second TSLP mRNA isoform (referred to as ‘short-form TSLP’) identified from normal human bronchial epithelial cells, whether two isoforms of TSLP (short-form and long-form TSLP) provide the identical function in the IFN-III/TSLP axis remains to be clarified (124). Moreover, when it comes to clinical application, it is currently unknown whether the IFN-III/TSLP axis is available to stimulate vaccine-induced antibody production in the human upper respiratory tract, as observed in mice. In addition, since IFNLR is specifically expressed on epithelial cells, the protective effect of the IFN-III/TSLP axis is exhibited particularly on mucosal sites through the intranasal and rectal vaccination way rather than the intraperitoneal or subcutaneous way. Therefore, mucosal vaccination seems to be the optimal choice for children and the elderly, as this vaccination method produces less pain and high compliance. Besides, *in vivo* studies have shown that IFN-III induces fewer inflammatory responses than IFN-I in the respiratory and gastrointestinal tracts (12, 14, 125, 126), while TSLPR has also been reported to regulate intestinal inflammation (127), the IFN-III/TSLP axis thereby exhibits many benefits when coming into application. Although there are currently no approved IFN-III drugs for use in humans, pre-clinical experiments have suggested the bright prospects of IFN-III for its fewer side effects compared with IFN-I. A phase IIb study has shown that pegylated IFN-λ1 treatment can produce a similar or improved immune response against chronic hepatitis C virus infection with fewer side effects when compared to IFN-I (128). Another phase IIb trial has indicated the benefits of pegylated IFN-III therapy in chronic hepatitis B patients by enhancing NK and CD8^+^ T cell response, which therefore impeded viral replication and antigen levels, while the treatment of IFN-I induced detrimental outcomes (129). Hence, IFN-III vaccination and its regulated IFN-III/TSLP axis are undoubtedly more efficient in future applications. Additionally, although the immunity benefits of IFN-III have been revealed, it is not yet clear whether the IFN-III/TSLP axis functions in defeating severe acute respiratory syndrome coronavirus 2 (SARS-CoV-2) in the context of coronavirus disease 2019 (COVID-19) pandemic (130–135). To date, various SARS-CoV-2 vaccines and recombinant vaccines are consecutively developed and applied in clinical practice (136–138). However, there still exist multiple challenges. On the one hand, the adjuvants of SARS-CoV-2 vaccines are limited and only contain aluminum salt-based adjuvants, emulsion adjuvants (MF9 and AS03), and toll-like receptor agonists. On the other hand, vaccines-induced immune response is restricted, usually leading to TH1 activation instead of TH2. Besides, current vaccines are inoculated via the intramuscular route, which may lack the protection of mucosal immune responses. In this review, we illuminate that IFN-III can target mucosal surfaces and provide adaptive immunoregulatory functions in vaccine administration via the IFN-III/TSLP axis. Therefore, IFN-III is a potential candidate for the SARS-CoV-2 vaccine adjuvant, which can promote mucosal immunity and help to establish a stronger immune defense line by supplementing adaptive immune responses, especially by activating mucosal immunity, thereby better combating coronavirus-caused respiratory infections. Obviously, IFN-III has great values in clinical research and application. Further experimental studies and clinical trials are required to explore the characteristics of IFN-III in the field of COVID-19 vaccines.

## Author contributions

Conceptualization, LC and SX; methodology, LC; software, SX and ZM; validation, LC, ZM and WQ; formal analysis, SX; investigation, WQ; resources, LC; data curation, WQ and SX; writing—original draft preparation, LC; writing—review and editing, WQ and WL; visualization, SX; supervision, WQ; project administration, LC; funding acquisition, ZM. All authors contributed to the article and approved the submitted version.
